# 

**Published:** 1881-03

**Authors:** 


CONTENTS.

| | PAGE. | | PAGE. |
|---|---|---|---|
| Diseases of the Kidneys | 79 | Breakfast Table Talk | 99 |
| Imaginary Diseases | 81 | About Love | 100 |
| Muscular Exercise | 82 | Tomatoes | 101 |
| Congestion | 84 | Difference Between Cousins | 103 |
| Poetry | 85 | The Genus Homo | 104 |
| The Foster-Brothers | 86 | Snow at Great Altitudes | 107 |
| Care of the Hair | 94 | The Power of Humbug | 109 |
| How to Keep Warm | 96 | Five Weeks in a Trance | 110 |
| Waking the Wrong Man | 97 | Cancer | 111 |
| My Old House (poetry) | 98 | A Cure for Sick-Headache | 112 |

